# Biomarkers of Exposure to Low Dietary Fumonisin, Deoxynivalenol, and Zearalenone Concentrations in Turkeys and Chickens

**DOI:** 10.3390/toxins18090371

**Published:** 2026-08-28

**Authors:** Elodie Lassallette, Philippe Guerre

**Affiliations:** 1 IHAP, Université de Toulouse, INRAE, ENVT, 31076 Toulouse, France; elodie.lassallette@envt.fr; 2 Olmix S.A., ZA du Haut du Bois, 56580 Bréhan, France

**Keywords:** fumonisins, deoxynivalenol, zearalenone, biomarkers, sphingolipids, oxidative damages, liver

## Abstract

*Fusarium* mycotoxins are major contaminants of human and animal feed and pose significant risks to health. A recent recommendation from the European Commission drastically decreased the maximum tolerated concentrations of fumonisins (FBs) and deoxynivalenol (DON) in poultry feed. The objectives of this study were to characterize the effects of a 14-day exposure to a diet containing 7.5 mg FB1 + FB2/kg, 2.5 mg DON/kg and 0.6 mg zearalenone (FDZ diet) in turkeys and chickens, and to assess the persistence of these effects following withdrawal of the contaminated diet. No clinical signs of toxicity were observed in either species, and markers of hepatic oxidative damage remained unchanged. In contrast, sphingolipidome alterations were detected, being more pronounced in turkeys than chickens and more persistent in the liver than in plasma. The C22–C24:C16 ratios measured across several sphingolipid (SL) classes emerged as sensitive biomarkers of FB exposure, whereas the sphinganine:sphingosine ratio (Sa:So) remained unchanged. Determination of the 90th percentile (P90) thresholds for SL ratios in unexposed animals provided good-to-excellent discrimination between exposed and unexposed animals. Notably, this conservative percentile-based approach detected FB exposure using the Sa:So ratio despite the absence of significant differences in group means. Multivariate analysis of the complete sphingolipidome provided the highest discriminatory power, distinguishing exposed from unexposed turkeys and chickens for up to four days after withdrawal of the FDZ diet. Finally, the biological significance of alterations in d18:1P, 18:0/2:0, 18:1/16:0, and dihydrosphingolipids induced by the FDZ diet is discussed.

## 1. Introduction

*Fusarium* mycotoxins frequently co-occur in cereals and maize worldwide, posing significant health risks to both humans and animals [1,2]. Among them, fumonisins (FBs)—particularly fumonisin B1 (FB1), the most abundant and toxic congener—deoxynivalenol (DON), and zearalenone (ZEN) are the most prevalent, with contamination levels ranging from a few µg/kg to several tens of mg/kg. In 2006, maximum guidance or regulatory levels for these mycotoxins have been established to protect animal health, and adverse effects have generally been reported for dietary concentrations exceeding these limits [3]. A recent recommendation from the European Commission drastically decreased the maximum tolerated concentrations of DON in poultry feed, whereas the maximum tolerated FB concentrations were reduced for poultry except turkeys and ducks [4]. The critical effects supporting these conclusions include alterations in intestinal morphology for both DON and FBs and hepatic oxidative stress for FBs [5,6]. Notably, these effects were reported at dietary concentrations substantially lower than those previously considered without biological impact in chickens and turkeys [7,8]. Intestinal morphology is influenced by numerous physiological and environmental factors, and non-dose-dependent responses have been described when *Fusarium* mycotoxins occur in combination [9]. Likewise, although FBs, DON, ZEN, and many other contaminants are known to induce oxidative stress at high exposure levels, reported effects at low doses remain inconsistent, and the factors underlying this variability are still poorly understood [10,11].

The identification of sensitive and specific biomarkers is therefore essential for improving the assessment of exposure to *Fusarium* mycotoxins and their biological effects in humans and animals. Biomarkers can be broadly classified into biomarkers of exposure, which measure the parent compounds or their metabolites in biological matrices, and biomarkers of effect, which reflect biological responses to contaminant exposure [12]. For FBs, the sphinganine-to-sphingosine (Sa:So) ratio is a well-established biomarker directly associated with the inhibition of ceramide synthase (CerS) by FB1 [13]. This ratio increases markedly following FB exposure and precedes the appearance of clinical signs of intoxication. Accordingly, EFSA recommends the Sa:So ratio as a biomarker for evaluating the efficacy of feed additives intended to reduce fumonisin exposure in animals [12]. Unfortunately, the Sa:So ratio was unaffected by low FB levels in turkey and chicken feed [14]. More recently, different studies conducted in chickens, turkeys and pigs at different levels of FB in feed, alone and in association with other *Fusarium* mycotoxins, have proposed the ratios of C22–C24 to C16 molecular species within different SL classes as complementary biomarkers of FB toxicity [15,16,17,18,19,20,21]. These include ceramides (Cers), sphingomyelins (SMs), monohexosylceramides (HexCers), and lactosylceramides (LacCers), derived from So, from Sa (dihydrosphingolipids, DHSLs), or from other sphingoid bases such as d18:2. These lipidomic biomarkers appear to combine high sensitivity and specificity for FB exposure. In contrast, although numerous biomarkers of effect have been investigated for DON and ZEN in poultry, none has yet demonstrated sufficient sensitivity and specificity to achieve broad consensus, particularly at low dietary exposure levels [22].

Health risk assessment of chemical contaminants generally relies on two complementary approaches [23,24,25]. The NOAEL and the LOAEL are derived from toxicological studies and identify, respectively, the highest dose producing no detectable adverse effects and the lowest dose at which adverse effects are observed. However, these estimates depend on the dose spacing selected in the experimental design and on the statistical power determined by sample size. An alternative approach is to use the 90th percentile (P90), which provides a robust estimate of the upper range of biological responses while minimizing the influence of extreme outliers. This metric may be particularly useful for evaluating biomarker responses under low-level mycotoxin exposure, where conventional toxicological endpoints are often difficult to detect [26]. Furthermore, because P90-based analyses compare response distributions rather than relying solely on mean differences, they may reduce the number of experimental groups and animals required, thereby supporting the 3R principle of reduction, provided that P90 is used as a complementary comparative metric rather than as a replacement for conventional toxicological endpoints.

The objective of the present study was to characterize the effects of dietary exposure to feed containing 7.5 mg/kg FB1 + FB2, 2.5 mg/kg DON, and 0.6 mg/kg ZEN (FDZ feed) in chickens and turkeys. This combined exposure was designed to reproduce a realistic contamination scenario, with FB and DON concentrations close to those recently associated with adverse effects in poultry. Biomarkers of hepatic oxidative stress and sphingolipid metabolism were evaluated to assess both general toxicological responses and FB-specific biological effects. To investigate the persistence of these responses, analyses were performed at the end of exposure and 2 and 4 days after withdrawal of the contaminated feed. Multiple statistical approaches were applied to characterize the observed biomarker responses.

## 2. Results

The effects of the FDZ diet on the performance and health of turkeys and chickens were reported previously, with particular emphasis on FB1 and ZEN as biomarkers of exposure [14]. No FDZ diet-related effects were observed on body weight, daily weight gain, feed conversion ratio, or the relative weights of the liver, heart, intestine, ceca, gizzard, bursa of Fabricius, pancreas, or spleen.

Similarly, hepatic MDA, GSH, and GSSG concentrations, as well as CAT activity, did not differ between groups (Table S1). Moreover, the 90th percentile (P90) values calculated for these parameters did not provide satisfactory discrimination between animals fed the FDZ diet and unexposed controls.

### 2.1. Alteration of the Sphingolipidome in Turkeys

#### 2.1.1. Hepatic Sphingolipidome

**Figure 1:**
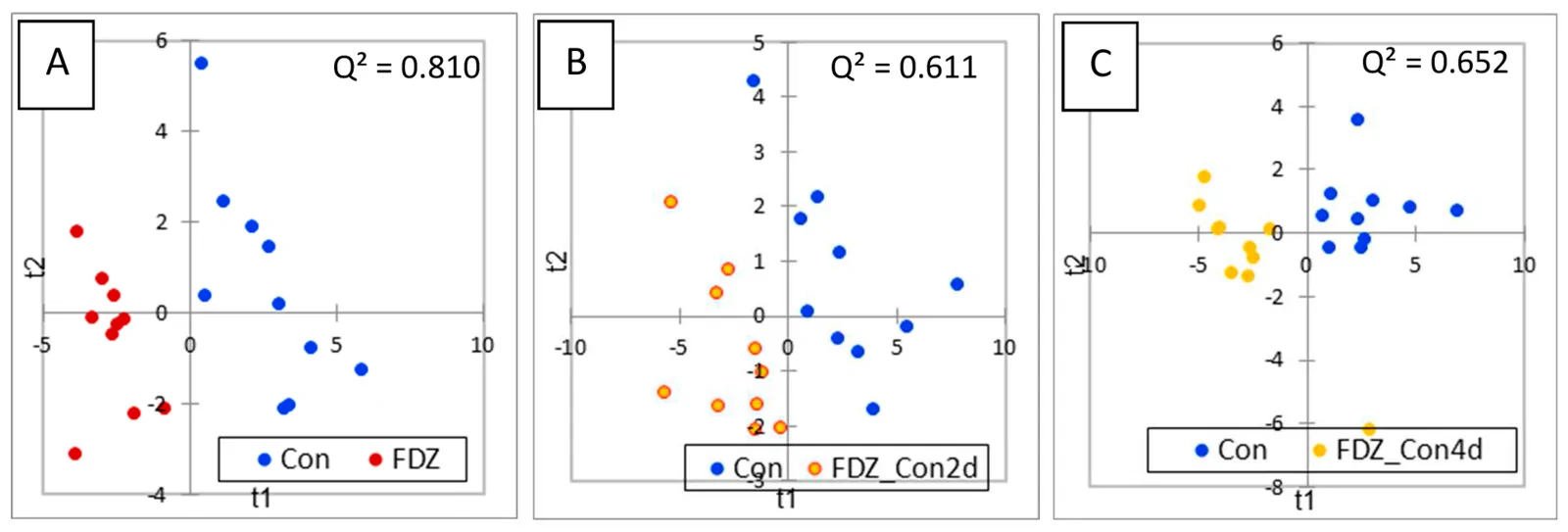
Partial least squares discriminant analysis (PLS-DA) of the hepatic sphingolipidome in turkeys (**A**) fed the control diet (Con) or the diet containing *Fusarium* mycotoxins (FDZ), (**B**) fed the Con diet or the FDZ diet followed by two days of the Con diet (FDZ_Con2d), and (**C**) fed the Con diet or the FDZ diet followed by four days of the Con diet (FDZ_Con4d). Q^2^ represents the predictive performance of the model. Sphingolipids with high variable importance in projection (VIP) scores are shown in bold in Table 1.

Partial least squares discriminant analysis (PLS-DA) of the hepatic sphingolipidome clearly discriminated turkeys fed the control (Con) diet from those receiving the *Fusarium* mycotoxin-contaminated (FDZ) diet, yielding a robust model with a Q^2^ value of 0.810 based on the first two components (Figure 1A). Interestingly, PLS-DA performed at 2 and 4 days of feeding the control diet continued to distinguish previously exposed turkeys from controls, with robust models exhibiting Q^2^ values of 0.611 and 0.652, respectively (Figure 1B,C).

The variables contributing most strongly to group separation corresponded to those showing significant difference between groups (Table 1). No significant changes were observed in the sphingoid bases Sa or So, whereas m18:0 levels were reduced. Likewise, sphingoid base derivatives were generally unaffected, with the exception of 18:0/2:0, which increased significantly.

The effects on other SL classes depended on acyl-chain length. C16-containing SLs, including Cer, HexCer, LacCer, and SM species, were generally decreased, and these reductions persisted for up to 4 days after withdrawal of the FDZ diet. In contrast, C22–C26 SL species exhibited more variable responses.

**Figure 2:**
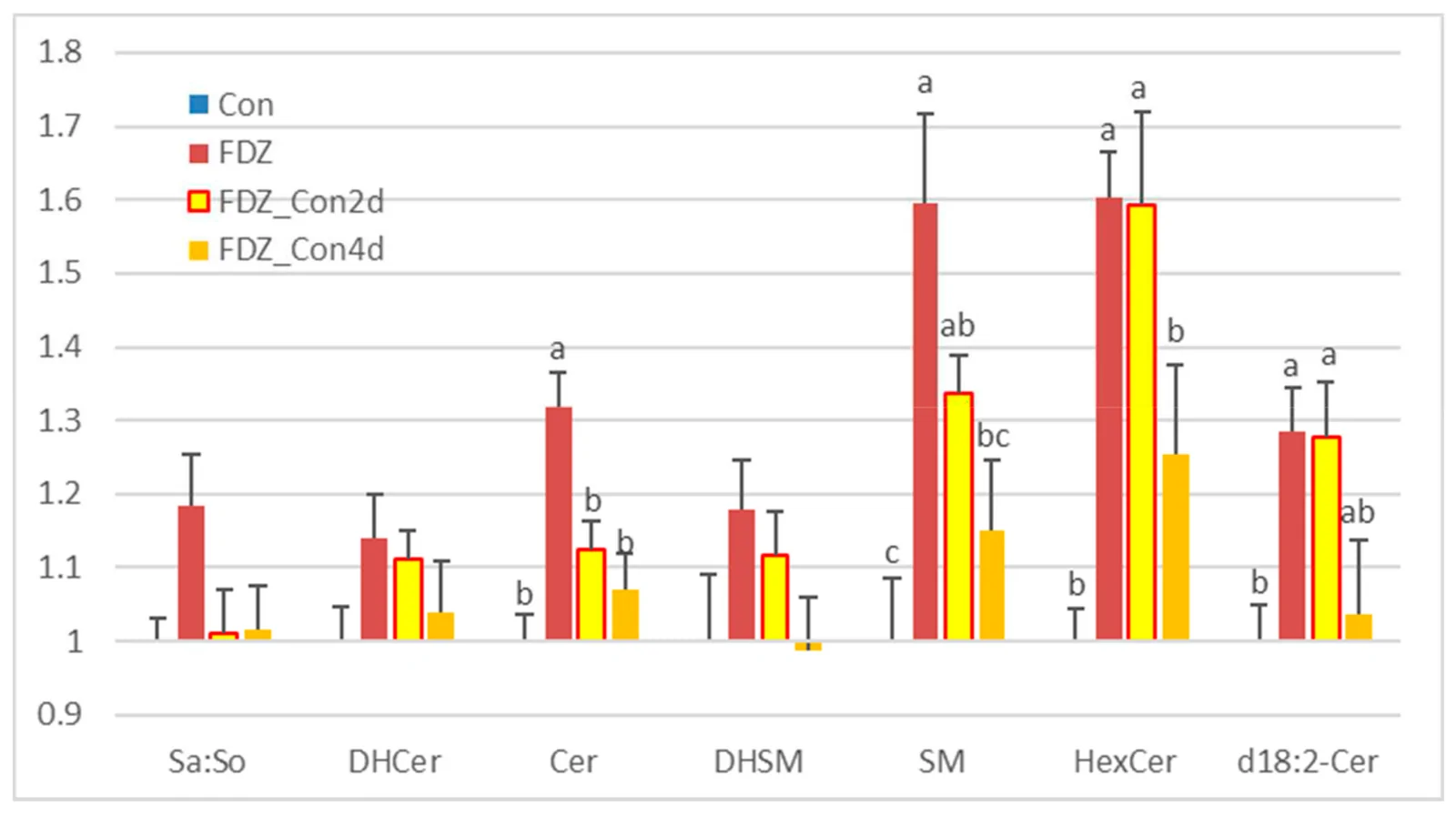
Hepatic Sa:So ratio and C22–C24:C16 sphingolipid ratios in turkeys fed the control diet (Con), the diet containing *Fusarium* mycotoxins (FDZ), the FDZ diet followed by two days of the Con diet (FDZ_Con2d), or the FDZ diet followed by four days of the Con diet (FDZ_Con4d). Values (*n* = 10 per group) are expressed as mean ± SE fold changes relative to the Con group. Groups were compared by one-way ANOVA. When a significant effect was detected (*p* < 0.05), pairwise comparisons were performed. Different letters denote statistically significant differences between groups (*p* < 0.05).

The Sa and C22–C24 ratios for the different SL classes are presented in Figure 2. Although the Sa ratio remained unchanged, the C22–C24 ratios for Cer, SM, HexCer, and d18:2-Cer increased following cessation of the FDZ diet. These increases remained significant after 2 days of feeding the control diet for SM, HexCer, and d18:2-Cer (Figure 2). The elevated C22–C24 ratios were primarily driven by reductions in C16 SL species (Table 1).

**Figure 3:**
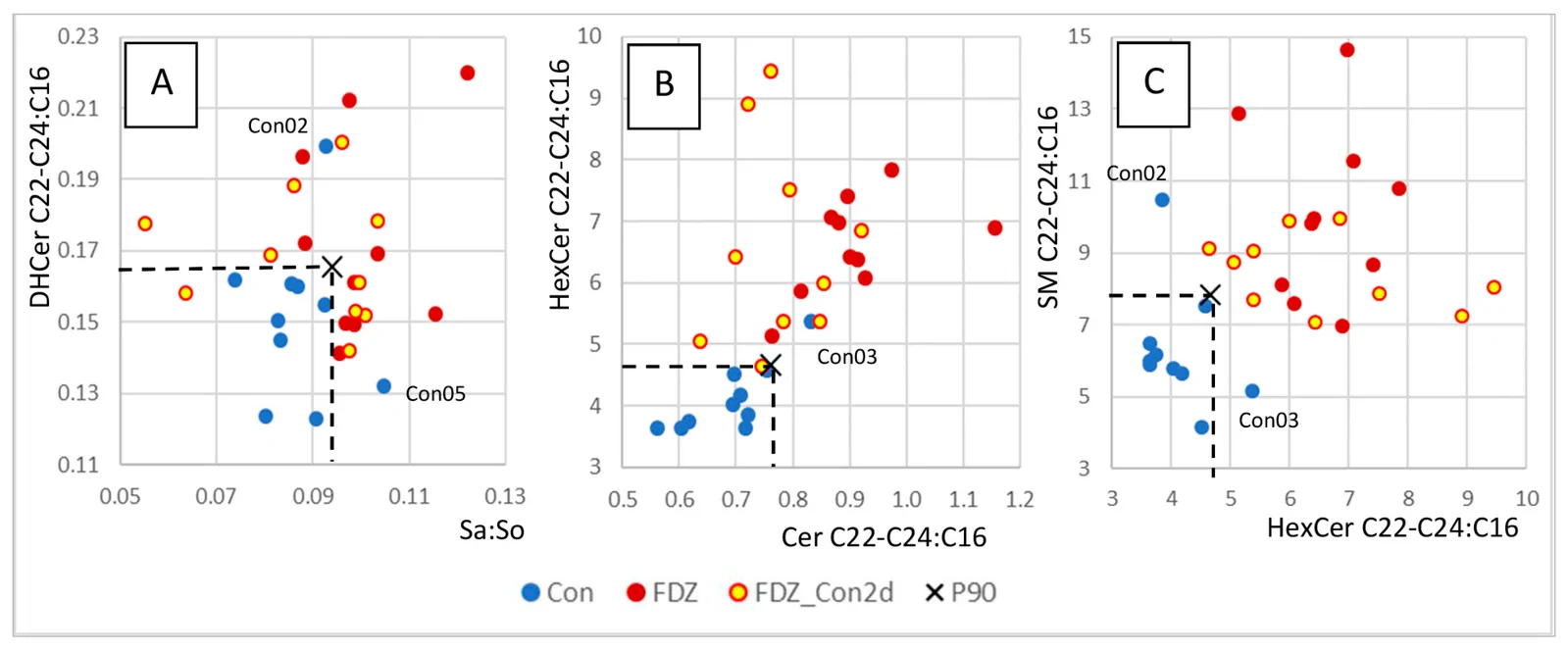
Distribution of selected hepatic sphingolipid ratios in turkeys fed the control diet (Con), the diet containing Fusarium mycotoxins (FDZ), the FDZ diet followed by two days of the Con diet (FDZ_Con2d), or the FDZ diet followed by four days of the Con diet (FDZ_Con4d). (**A**) C22–C24:C16 ratio of DHCer versus the Sa:So ratio; (**B**) C22–C24:C16 ratio of HexCer versus C22–C24:C16 ratio of Cer; (**C**) C22–C24:C16 ratio of SM versus C22–C24:C16 ratio of HexCer. Dotted lines indicate the estimated 90th percentile (P90) values calculated from the Con group.

To evaluate the potential of SL ratios as biomarkers of FB exposure, the P90 values determined in unexposed turkeys were calculated and compared with individual values from control and FDZ-exposed turkeys at the end of the exposure period and after 2 days of feeding the control diet (Table 2, Figure 3). The combination of different P90 thresholds provided good discrimination between exposed and unexposed animals (Figure 3). ROC curve analysis was also used to assess the distribution of the animals in the different groups (Table 2). Using this analysis, the thresholds for the hepatic C22–C24:C16 ratios of SM and HexCer provided the highest sensitivity, with specificities of 80 and 90%, respectively (Table 2). In contrast, the Sa:So ratio and the C22–C24:C16 ratio of DHCer showed low sensitivity whereas the specificity was high. Finally, this analysis revealed that the highest accuracy was obtained with the C22–24:C16 ratios measured for the HexCer and SM. Accuracies of 70 and 75% were also obtained for the C22–24:C16 ratios measured for the DHSM and Cer, respectively.

Overall, feeding turkeys the FDZ diet induced extensive alterations in the hepatic sphingolipidome. Besides changes in the Sa:So and C22–C24:C16 ratios across several SL classes, the most prominent alterations include increased 18:0/2:0 concentrations and decreased 18:1/16:0 concentrations, both of which persisted after withdrawal of the FDZ diet. Multiple analytical approaches successfully distinguished exposed from unexposed animals. Notably, the P90 threshold of the Sa:So ratio enabled discrimination between groups despite the absence of significant differences in mean values.

#### 2.1.2. Plasma Sphingolipidome

**Figure 4:**
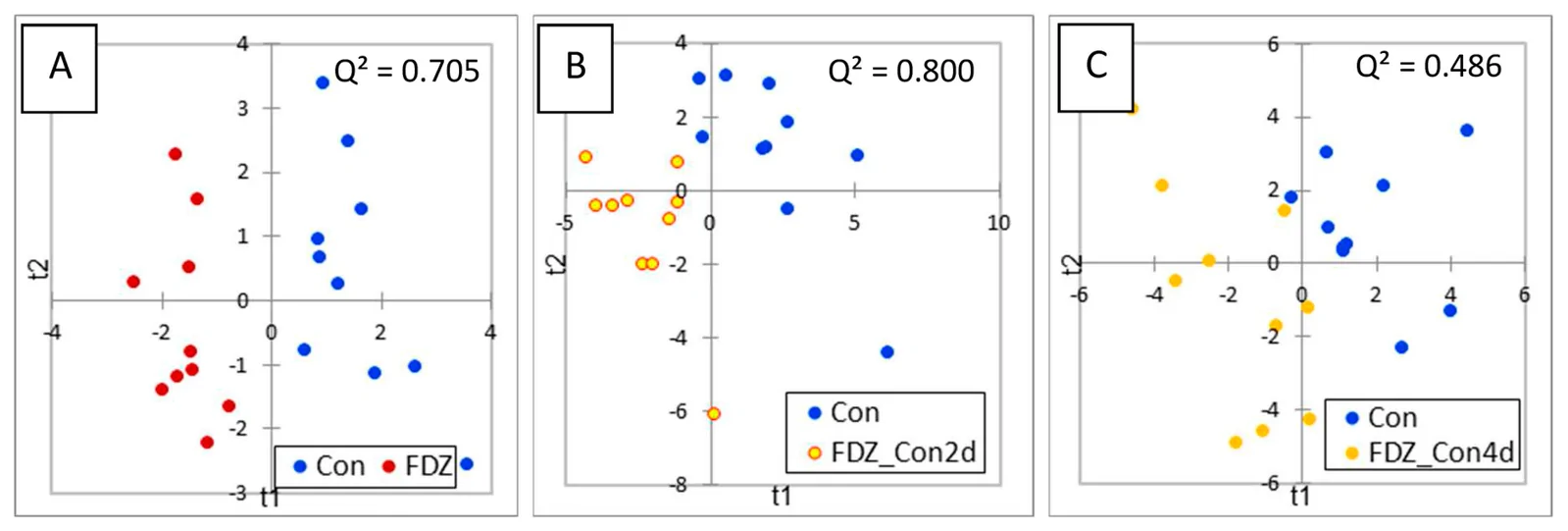
Partial least squares discriminant analysis (PLS-DA) of the plasma sphingolipidome in turkeys (**A**) fed the control diet (Con) or the diet containing *Fusarium* mycotoxins (FDZ), (**B**) fed the Con diet or the FDZ diet followed by two days of the Con diet (FDZ_Con2d), and (**C**) fed the Con diet or the FDZ diet followed by four days of the Con diet (FDZ_Con4d). Q^2^ represents the predictive performance of the model. Variables with high variable importance in projection (VIP) scores are shown in bold in Table 3 and Table S2.

PLS-DA of plasma SLs measured at the end of the exposure period discriminated control turkeys from those fed the FDZ diet within a robust model, with a Q^2^ value of 0.705 (Figure 4A). Similarly, after two days of feeding the control diet, previously exposed turkeys remained clearly distinguishable from controls in a robust model (Q^2^ = 0.800; Figure 4B). After four days of feeding the control diet, discrimination between the two groups was still possible; however, the lower Q^2^ value (0.486) indicated limited model robustness (Figure 4C).

Plasma concentrations of individual SLs, total SLs by class, the Sa:So and Sa1P:So1P ratios, and the C22–24:C16 ratios are presented in Table S2. ANOVA revealed that only a limited number of variables were significantly affected by the FDZ diet. Although sphingoid bases and their phosphorylated derivatives exhibited variable responses, phosphorylated species generally showed a trend toward increased concentrations (Table 3). In contrast, 18:0/2:0 concentrations increased significantly upon withdrawal of the FDZ diet, and this effect persisted throughout the recovery period (Table 3). Conversely, 18:1/16:0 concentrations decreased following cessation of the FDZ diet, with the reduction persisting after two days of feeding the control diet but no longer evident after four days. Transient, non-significant increases were also observed in the C22–24:C16 ratios of Cer and HexCer (Table S2).

The P90 values of the Sa:So and C22–C24:C16 ratios were subsequently evaluated as potential biomarkers in plasma. However, neither ratio provided satisfactory discrimination between exposed and unexposed turkeys.

Overall, alterations in the plasma sphingolipidome were less pronounced than those observed in the liver. The principal changes associated with the FDZ diet were a sustained increase in 18:0/2:0 concentrations and a transient decrease in 18:1/16:0 concentrations. Neither the Sa:So nor the C22–C24:C16 ratios discriminated exposed from unexposed animals, even when P90 thresholds were applied. In contrast, PLS-DA of the complete plasma sphingolipidome successfully distinguished turkeys fed the FDZ diet from those fed the control diet.

### 2.2. Alteration of the Sphingolipidome in Chicken

#### 2.2.1. Hepatic Sphingolipidome

**Figure 5:**
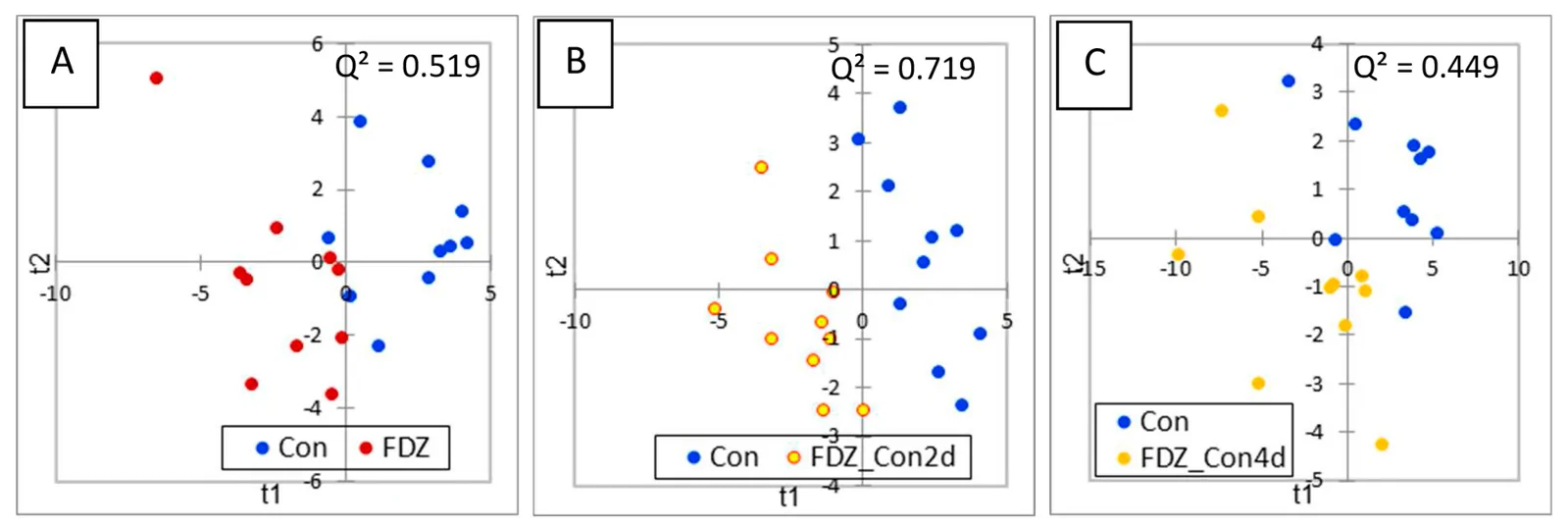
Partial least squares discriminant analysis (PLS-DA) of the hepatic sphingolipidome in chickens (**A**) fed the control diet (Con) or the diet containing *Fusarium* mycotoxins (FDZ), (**B**) fed the Con diet or the FDZ diet followed by two days of the Con diet (FDZ_Con2d), and (**C**) fed the Con diet or the FDZ diet followed by four days of the Con diet (FDZ_Con4d). Q^2^ represents the predictive performance of the model. Variables with high variable importance in projection (VIP) scores are shown in bold in Table 4 and Table S3.

PLS-DA of the hepatic sphingolipidome discriminated chickens fed the Con diet from those receiving the FDZ diet, yielding a model with a Q^2^ of 0.519 (Figure 5A). After two days of feeding the Con diet, previously exposed chickens remained distinguishable from controls within a robust model (Q^2^ = 0.719; Figure 5B). After four days of feeding the Con diet, separation between the two groups was still achieved; however, the lower Q^2^ value (0.449) indicated limited model robustness (Figure 5C).

Hepatic SL concentrations are presented in Table S3, and the variables showing significant differences between groups are summarized in Table 4. Among the observed changes, Sa and So concentrations increased following withdrawal of the FDZ diet, and these elevations persisted after two and four days of feeding the Con diet. Most d18:1 Cer species remained unchanged throughout the study. In contrast, Cer species derived from the sphingoid bases d18:0, m17:0, m18:1, d18:2, and t18:0 generally increased, with the strongest effects frequently observed after two days of feeding the control diet following FDZ exposure.

A transient decrease in Hex18:1/18:0 and Lac18:1/20:0 concentrations was also observed upon cessation of the FDZ diet. In addition, SM concentrations generally showed a non-significant upward trend in chickens switched from the FDZ diet to the Con diet.

**Figure 6:**
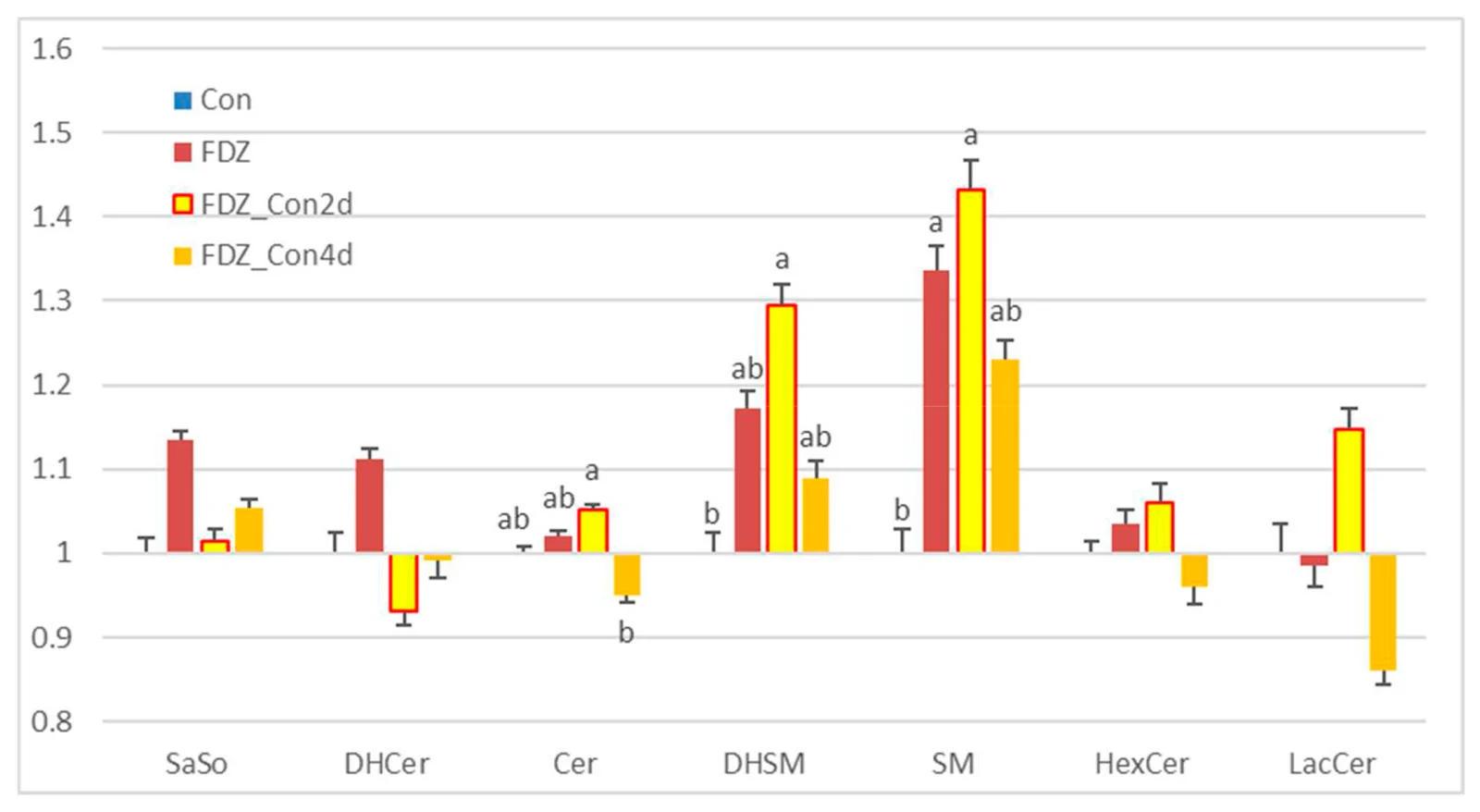
Hepatic Sa:So and C22–C24:C16 sphingolipid ratios in chickens fed the control diet (Con), the diet containing *Fusarium* mycotoxins (FDZ), the FDZ diet followed by two days of the Con diet (FDZ_Con2d), or the FDZ diet followed by four days of the Con diet (FDZ_Con4d). Values (*n* = 10 per group) are expressed as mean ± SE fold changes relative to the Con group. Groups were compared by one-way ANOVA. When a significant effect was observed (*p* < 0.05), pairwise comparisons were performed. Different letters denote statistically significant differences between groups (*p* < 0.05).

No significant changes in the Sa:So ratio were observed in the liver (Figure 6). In contrast, the C22–24:C16 ratios of DHSM and SM increased following withdrawal of the FZ diet and remained elevated after two days of feeding the Con diet (Figure 6). The corresponding ratios for the other SL classes showed only minor and inconsistent changes.

The P90 values of the Sa:So and C22–C24:C16 ratios were subsequently evaluated as potential biomarkers to distinguish exposed from unexposed chickens (Table 2). Complementary ROC curve analysis provided partial discrimination between the two groups. The best sensitivities were obtained for the C22–C24:C16 ratios measured for the SM and DHCer. Conversely, the C22–24:C16 ratio for the HexCer showed excellent specificity but low sensitivity. Finally, an accuracy of 65–78% was achieved using the C22–C24:C16 ratios measured for the DHSM, DHCer, and SM.

Overall, alterations in the hepatic sphingolipidome of chickens enabled discrimination between exposed and unexposed animals using both multivariate and ratio-based approaches. In addition to PLS-DA of the complete sphingolipidome, combinations of P90 thresholds derived from selected SL ratios provided satisfactory discrimination at the end of the FDZ feeding period and after two days of feeding the Con diet.

#### 2.2.2. Plasma Sphingolipidome

**Figure 7:**
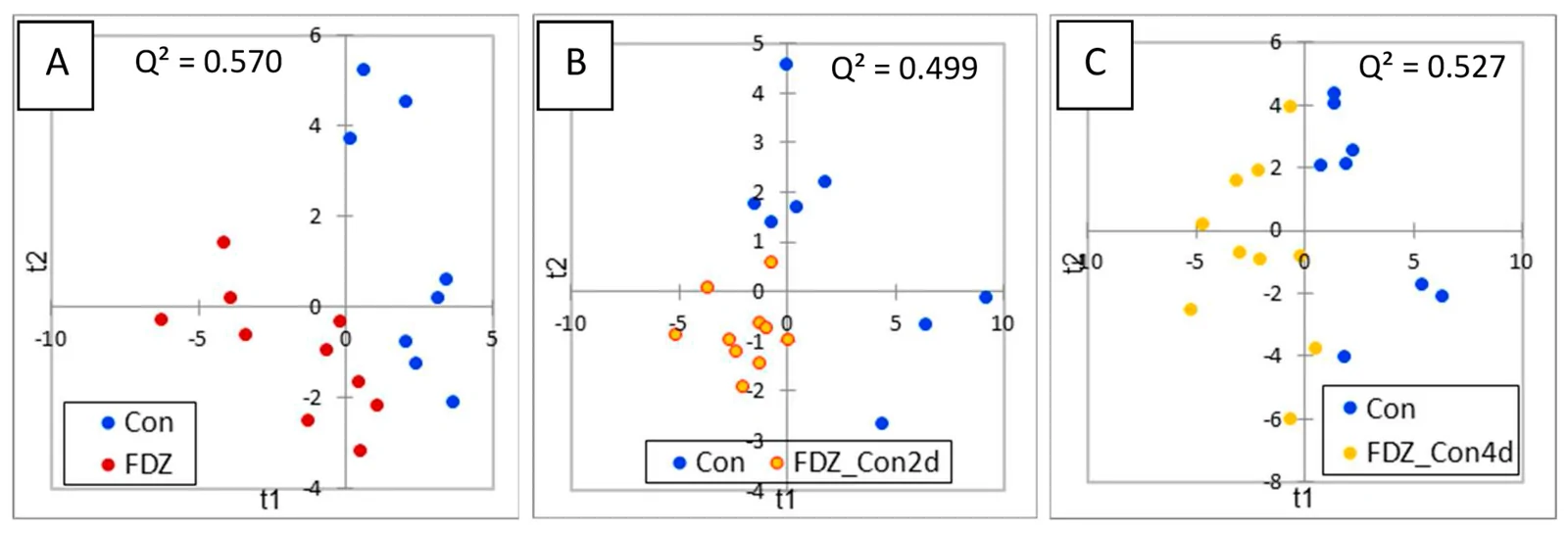
Partial least squares discriminant analysis (PLS-DA) of the plasma sphingolipidome in chickens (**A**) fed the control diet (Con) or the diet containing *Fusarium* mycotoxins (FDZ), (**B**) fed the Con diet or the FDZ diet followed by two days of the Con diet (FDZ_Con2d), and (**C**) fed the Con diet or the FDZ diet followed by four days of the Con diet (FDZ_Con4d). Q^2^ represents the predictive performance of the model. Variables with high variable importance in projection (VIP) scores are shown in bold in Table 5 and Table S4.

PLS-DA of the plasma sphingolipidome discriminated chickens fed the Con diet from those receiving the FDZ diet, yielding a model with a Q^2^ value of 0.570 (Figure 7A). After two and four days of feeding the Con diet, previously exposed chickens remained distinguishable from controls; however, the respective Q^2^ values of 0.499 and 0.527 indicated limited model robustness (Figure 7B,C).

Plasma SL concentrations are presented in Table S4. Overall, only a limited number of plasma SLs were significantly affected by the FDZ diet (Table 5). Following withdrawal of the FDZ diet, concentrations of d18:1, d18:0P, and d18:1P increased significantly. Similarly, very-long-chain DHCer species and total DHCer concentrations increased upon cessation of FDZ exposure before declining after feeding the Con diet. Most Cer species exhibited variable responses, with the most pronounced effect being a decrease in concentrations after 2–4 days of feeding the Con diet (Table 5). Neither the Sa:So nor the Sa1P:So1P ratio differed significantly between groups (Table S4). The C22–C24:C16 ratio of Cer increased following withdrawal of the FDZ diet and returned toward control values after four days of feeding the Con diet (Table 5), whereas the corresponding ratios for the other SL classes remained largely unchanged.

The P90 values of the Sa:So ratio and C22–C24:C16 ratios were subsequently evaluated as potential plasma biomarkers. However, none of these ratios provided satisfactory discrimination between exposed and unexposed chickens.

Overall, alterations in the plasma sphingolipidome of chickens were modest and depended on the duration of the recovery period following FDZ exposure. Although PLS-DA distinguished chickens fed the FDZ diet from controls, the robustness of the resulting models was limited. In contrast, neither the Sa:So nor the C22–C24 ratios provided satisfactory discrimination between exposed and unexposed animals.

## 3. Discussion

Identifying biomarkers of mycotoxin-induced effects remains a major challenge in both human and animal health. Sensitive and specific biomarkers are particularly valuable in animal nutrition for evaluating the efficacy of feed additives intended to reduce the impact of mycotoxin contamination. Because these efficacy studies must be conducted using mycotoxin concentrations below the maximum levels recommended for animal feed [27], identifying variables that can serve as reliable positive controls is often difficult. Moreover, a recent recommendation from the European Commission drastically decreased the maximum tolerated concentrations of fumonisins (FBs) and deoxynivalenol (DON) in poultry feed.

In the present study, the FDZ diet did not induce significant changes in markers of hepatic oxidative damage in either chickens or turkeys. These findings are consistent with previous studies conducted in both species at higher exposure levels [7,8]. However, they contrast with a previous study reporting that dietary FB concentrations close to 5 mg FB1 + FB2/kg feed induced hepatic oxidative damage in chickens FBs [6]. Potential explanations for this discrepancy are discussed below.

Quantification of sphingoid bases by LC-MS/MS, together with calculation of the Sa:So ratio, revealed no significant differences between treatment groups in the liver of either turkeys or chickens. These findings are consistent with our previous measurements of sphingoid bases in the same animals using HPLC with fluorometric detection [14]. Likewise, no significant increase in the Sa1P:So1P ratio was observed, although a transient decrease was detected in turkey plasma two days after withdrawal of the FDZ diet. The Sa:So and Sa1P:So1P ratios are well-established biomarkers of fumonisin-induced inhibition of CerS across numerous animal species [13,16]. Among these, the plasma Sa1P:So1P ratio is generally considered more sensitive than the Sa:So ratio for detecting FB exposure [15,17,19,28,29]. Moreover, both ratios are generally unaffected by the concomitant presence of other *Fusarium* mycotoxins. The absence of an increase in either ratio in the present study therefore most likely reflects the low FB concentration in the experimental diet. Despite the lack of changes in these ratios, plasma concentrations of 18:1P and, to a lesser extent, d18:0P increased in both species. Increased So1P concentrations may reflect a mild inflammatory response induced by *Fusarium* mycotoxins [18,30]. Indeed, S1P is a key regulator of immune and inflammatory processes, acting through S1P receptor-mediated signaling pathways to promote and amplify inflammatory responses [31,32].

Inflammation is also closely linked to oxidative stress, as excessive inflammatory responses increase the production of reactive oxygen species while disrupting the balance between pro-oxidant and antioxidant mechanisms, thereby contributing to hepatic oxidative damage [33]. In the present study, the inflammatory response appears to have been insufficient to induce measurable oxidative damage in the liver. Conversely, a more pronounced inflammatory response may explain the hepatic oxidative damage reported previously in chickens exposed to similar FB concentrations [6]. Unfortunately, SLs were not measured in that earlier study, preventing direct evaluation of this hypothesis. Disruption of sphingolipid metabolism serves as both a marker and a potential mechanism of response to toxic stress; however, the resulting increase is not necessarily accompanied by a parallel activation of pro-inflammatory cytokine production. Sphingolipids and cytokines operate via distinct signaling pathways that are highly dependent on cell type, subcellular compartment, and exposure kinetics. A recent study on the effects of fumonisins in chickens revealed changes in the oxylipidome and sphingolipidome suggestive of a mild inflammatory response, yet without any rise in pro-inflammatory cytokine concentrations [30]. Furthermore, an in vitro study on chicken splenic lymphocytes demonstrated a decrease in cytokines [34]—an effect consistent with the immunomodulatory impact of FBs reported in various reviews [35,36].

Several ratios between very-long-chain (C22–C24) and long-chain (C16) SLs increased following feeding of the FDZ diet. In turkeys, the hepatic C22–C24:C16 ratios of Cer, SM, HexCer, and d18:2-Cer increased after withdrawal of the FDZ diet, and these changes persisted for two days after the animals were switched to the uncontaminated Con diet. In chickens, increased hepatic C22–C24:C16 ratios were observed for DHSM and SM, whereas a significant increase in the C22–C24:C16 ratio of Cer was detected in plasma. These changes were primarily driven by a reduction in C16 SL level species, consistent with previous studies identifying C22–C24:C16 ratios as sensitive biomarkers of FB exposure [16]. Although mono-unsaturated Cer species decreased in turkeys during the recovery period, this effect was not observed in other SL classes and did not prevent an increase in the hepatic C22–C24:C16 ratio of Cer. The changes in C22–C24:C16 ratios were attributed to the presence of FBs in the FDZ diet. Indeed, previous studies conducted at contamination levels close to the recommended maximum concentrations for FB, DON, and ZEN showed that, although interactions among *Fusarium* mycotoxins affected the sphingolipidome, they did not alter the effects of FBs on C22–C24:C16 ratios [7,8]. Collectively, these findings demonstrate that the C22–C24:C16 ratios measured in several SL classes, particularly Cer, HexCer, and SM, are highly sensitive indicators of exposure to low dietary FB concentrations.

The P90 values of the Sa:So and C22–C24:C16 ratios were determined in the liver and plasma of turkeys and chickens. Comparing these thresholds established in unexposed animals with the corresponding values obtained in exposed animals has previously been proposed as a robust approach for identifying toxicant exposure [23,24,25,26]. In the present study, this approach did not provide satisfactory discrimination between exposure groups when applied to plasma. In contrast, hepatic measurements successfully distinguished exposed from unexposed animals in both species. In turkeys, the highest accuracy was achieved with the C22–C24:C16 ratio of HexCer, followed by SM and Cer, whereas in chickens the SM ratio showed the greatest discriminatory power, followed by DHCer and DHSM. The hepatic Sa:So ratio also contributed to the discrimination of exposed animals in both species. These findings highlight the potential value of a percentile-based approach as a complement to conventional comparisons of group means when assessing the efficacy of feed additives intended to mitigate mycotoxin contamination.

The increases in C22–C24:C16 ratios observed in this study were generally driven by reductions in C16-SL concentrations, in agreement with previous reports obtained at higher FB exposure levels [15,16]. This observation is particularly relevant because C16-Cer and C22–24-Cer are synthesized predominantly by CerS5/6 and CerS2, respectively [37]. Beyond the differential sensitivity of CerS isoforms to FB inhibition, these changes may also have toxicological implications. In cell models, accumulation of C16 has been associated with pro-apoptotic effects, whereas C24 exert anti-apoptotic functions [38]. Consistent with these findings, very-long-chain Cers have been shown to attenuate C16-induced permeabilization of rat liver mitochondria [39]. Moreover, the sphingolipid alterations observed in CerS2-deficient mice closely resemble those induced by FB_1_ exposure in mammals and are associated with hepatic toxicity [40,41,42].

However, although the hepatic C22–C24:C16 ratio of DHCer showed a numerical increase in chickens fed the FDZ diet, this increase was not driven by a reduction in C16 DHCer species, as observed for other SL classes. Instead, DHCer species of all chain lengths increased in chicken liver. This finding is consistent with previous studies in chickens and turkeys reporting a complex response of DHSLs to FB exposure. Together, these observations indicate that changes in DHCer and DHSM, as well as their corresponding C22–C24 ratios, should be interpreted with caution when assessing the effects of very low FB exposure. A similar conclusion applies to chicken plasma. Although several significant changes in plasma SL concentrations were detected, these alterations do not conform to the established mechanisms of FB toxicity, and their biological significance remains uncertain.

Another noteworthy finding was the marked increase in 18:0/2:0 concentrations in both the liver and plasma of turkeys fed the FDZ diet. This observation is consistent with previous studies conducted at higher dietary FB concentrations [8,15,17,30], and is of particular interest given the potential biological activity of 18:0/2:0 [31]. Although the underlying mechanisms remain speculative, the concurrent increases in 18:0/2:0, DHCer, and DHSM concentrations observed here, as well as in previous studies, may reflect differences in the affinity of N-acetyltransferases and CerS for Sa, So, and acetyl- or acyl-CoA substrates [43]. Preferential metabolism of Sa could therefore account for the accumulation of 18:0/2:0, DHCer, and DHSM while simultaneously explaining the absence of a detectable increase in the Sa:So ratio.

Ultimately, multivariate analysis of the complete sphingolipidome provided the highest discriminatory power for distinguishing animals fed the FDZ diet from animals fed unexposed controls. This finding underscores that the effects of FBs on sphingolipid metabolism extend well beyond alterations in the Sa:So ratio. Although model robustness declined after withdrawal of the contaminated diet, PLS-DA continued to distinguish exposed turkeys and chickens from controls after four days of feeding the uncontaminated diet. This persistence is consistent with the relatively slow elimination of FB1 from the liver, with reported hepatic half-lives of approximately 124 h in turkeys and 66 h in chickens [14]. More generally, these results also demonstrate that turkeys are more sensitive than chickens to the effects of FBs on the sphingolipidome. This observation is consistent with previous research conducted on sphingoid bases and their phosphorylated forms at higher doses [44]. It also aligns with earlier studies on the general toxicity of FBs in these two species [45,46,47].

## 4. Conclusions

In conclusion, this study is the first to compare different statistical approach for detecting the effects of very-low-level exposure to *Fusarium* mycotoxins in turkeys and chickens at both hepatic and plasma levels. Biomarkers were more altered in turkeys than in chickens. In both species, clear alterations of the sphingolipidome were observed. These changes were generally more pronounced and more persistent in the liver than in plasma, with notable species-specific differences, particularly in the regulation of dihydrosphingolipids. The most consistent response was an increase in the C22–C24:C16 ratios across several sphingolipid classes. Determination of the P90 thresholds for these ratios in unexposed animals provided good-to-excellent discrimination between exposed and unexposed animals in both species. Although the number of animals used in the various groups was relatively small, the value of this approach was particularly evident for the Sa:So ratio, for which percentile-based analysis detected exposure despite the absence of significant differences in mean values. Multivariate analysis of the complete sphingolipidome remained the most sensitive approach for distinguishing exposed from unexposed animals in both liver and plasma. Significant increase in the concentrations of 18:0/2:0, 18:1P, DHCer, and DHSM suggested a mild inflammatory response, the intensity of which remained sufficiently controlled to prevent liver oxidative damages. Further studies are needed to determine whether interactions between *Fusarium* mycotoxins at the tested dosage levels result in independent or interactive effects.

## 5. Materials and Methods

### 5.1. Analytes and Reagents

Analytical standards and reagents were purchased from Sigma-Aldrich (Sigma-Aldrich Chimie SARL, Saint-Quentin-Fallavier, France) and Scharlab (Scharlab S.L., Sentmenat, Spain). Reagents and solvents used for SL analysis were of LC-MS/MS grade, whereas those used for analyte extraction and oxidative damage assays were of HPLC grade. SL external standards were purchased from Avanti Polar Lipids (Avanti Polar Lipids, Alabaster, AL, USA). The complete list of external standards has been reported previously in [15]. Internal standards consisted of the Ceramide/Sphingoid Internal Standard Mixture I, containing C17-sphingosine, C17-sphinganine, C17-sphingosine-1-phosphate, C17-sphinganine-1-phosphate, C12:0-lactosyl(β)-ceramide, C12:0-sphingomyelin, C12:0-glucosyl(β)-ceramide, C12:0-ceramide, C12:0-ceramide-1-phosphate, and C25:0-ceramide, supplemented with m17:1/12:0, m17:0/12:0, m18:1/12:0, and C12:0-ceramide sulfate. All internal standards were dissolved in ethanol at a final concentration of 25 μM.

### 5.2. Feed Preparation, Animal Husbandry, and Sampling

Corn–soybean-based diets were formulated to meet the nutritional requirements of each species [14]. Two batches of maize, one free of *Fusarium* mycotoxins and one naturally contaminated, were used to produce the control (Con) and contaminated (FDZ) diets, respectively. Concentrations of *Fusarium* mycotoxins and other potentially interfering mycotoxins were determined by HPLC-MS/MS [48] and are reported in Table 6.

All experimental procedures were conducted in accordance with French regulations governing the care and use of animals for scientific purposes. Feed and water were provided ad libitum throughout the study. Forty male Grade Maker turkeys and forty male Ross PM3 broiler chickens were reared on floor pens at the experimental facilities of the PEAT Unit (INRAe, Val de Loire Research Center, Nouzilly, France) under standard husbandry conditions. Birds received the uncontaminated diet until 55 days of age (turkeys) and 20 days of age (chickens). Four homogeneous groups of ten animals were constituted for each species at 48 days of age (turkeys) and 17 days of age (chickens). Feeding with the Con or FDZ diets started at 55 and 20 days of age in turkeys and chickens, respectively. After 14 days of exposure (69 days of age in turkeys and 34 days in chickens), one FDZ group and the corresponding control group were euthanized following electronarcosis and exsanguination. The remaining birds previously fed the FDZ diet were switched to the control diet for either 2 or 4 days before slaughter at 71 or 73 days of age in turkeys and 36 or 38 days of age in chickens. Body weight, feed intake, and zootechnical performance were recorded at each sampling time (55, 69, 71, and 73 days in turkeys; 20, 34, 36, and 38 days in chickens). At necropsy, all birds were examined for macroscopic lesions. Organs were excised, weighed, and the liver was immediately frozen at −80 °C until analysis.

### 5.3. Determination of Hepatic Oxidative Damage Markers

Liver samples (5 g) were homogenized at 4 °C in 15 mL of phosphate buffer (0.1 M, pH 7.4) containing Tris-acetate (0.1 M), potassium chloride (0.1 M), EDTA (1 mM), and butylated hydroxytoluene (0.02 M). The supernatant fraction (S9) was collected after centrifugation at 9000× *g* for 30 min. An aliquot (500 µL) was deproteinized using 1.25 M metaphosphoric acid (*v*/*v*) for glutathione measurement. Protein concentration was measured using the Bio-Rad Protein Assay Kit (Bio-Rad Laboratories, Munich, Germany). The S9 fraction and deproteinized extracts were stored at −80 °C until analysis.

Malondialdehyde (MDA) concentrations were determined fluorometrically (excitation 515 nm, emission 548 nm) after reaction with thiobarbituric acid [49], followed by butanol extraction [50,51]. Quantification was performed using calibration curves prepared with MDA standards.

Total glutathione and oxidized glutathione (GSSG) were determined by the enzymatic recycling method described by Baker et al. [52]. Reduced glutathione (GSH) concentrations were calculated as the difference between total glutathione and GSSG. Briefly, GSH reacts with 5,5′-dithiobis(2-nitrobenzoic acid) to form 5-thio-2-nitrobenzoic acid (TNB) and GS-TNB. Both GSSG and GS-TNB are subsequently reduced by glutathione reductase, allowing continuous TNB formation, which was monitored spectrophotometrically at 405 nm. Total glutathione was measured after a 1:100 dilution of the deproteinized extract in 0.2 M MES buffer (pH 6.0) containing 0.05 M K_2_HPO_4_ and 1 mM EDTA. GSSG was determined after derivatization of GSH with 2-vinylpyridine [53]. Concentrations were calculated using GSH and GSSG standard curves.

Catalase (CAT; EC 1.11.1.6) activity was measured in the S9 fraction (10 µg protein/mL) after incubation for 2.5 min at room temperature. The assay was based on the oxidation of methanol to formaldehyde in the presence of hydrogen peroxide, followed by reaction of formaldehyde with Purpald reagent to generate a chromophore quantified at 540 nm [54]. Enzyme activity was calculated from formaldehyde calibration curves.

### 5.4. Sphingolipidomic Analysis

Hepatic and plasma sphingolipids were quantified by LC-MSMS as previously described [15]. Briefly, 1 g of liver was homogenized in 3 mL of phosphate buffer (0.1 M, pH 7.4) and centrifuged at 3000× *g* for 15 min. An aliquot of 40 µL of the supernatant, or 40 µL of plasma, was diluted with 120 μL of 0.9% NaCl and spiked with internal standards to obtain a final concentration equivalent to 6250 pmol/g liver. Samples were extracted with 600 µL methanol/chloroform (2:1, *v*/*v*) and incubated overnight at 48 °C. After cooling, 100 μL of 1 M methanolic KOH was added to hydrolyze glycerophospholipids, followed by incubation for 2 h at 37 °C. The reaction was neutralized with 10 µL of 50% acetic acid, and SLs were extracted twice with 600 µL methanol/chloroform (2:1, *v*/*v*). Combined supernatants were evaporated to dryness, and residues were reconstituted in 200 μL methanol before LC-MS/MS analysis.

Chromatographic separation was performed on a Poroshell 120 column (3.0 × 50 mm, 2.7 µm) using an Agilent 1260 binary pump (Agilent Technologies, Santa Clara, CA, USA). Detection was carried out in positive electrospray ionization mode using dynamic multiple reaction monitoring (MRM) on an Agilent 6410 triple quadrupole mass spectrometer. MRM transitions, fragmentor voltages, collision energies, retention times, and method validation parameters have been reported previously [15]. Data were processed using Agilent MassHunter Quantitative Analysis software B.05.291.0. SLs lacking authentic standards were quantified using calibration curves from structurally related compounds with similar molecular masses. Final concentrations were corrected according to the recovery of the corresponding internal standards.

### 5.5. Statistical Analysis

Statistical analyses were performed using XLSTAT Biomed software 2018.1.1 62926 (Addinsoft, Bordeaux, France). Differences were considered statistically significant at *p* < 0.05. Global alterations in the hepatic sphingolipidome induced by the FDZ diet were investigated by partial least squares discriminant analysis (PLS-DA). Variables with a variable importance in projection (VIP) score > 1 were considered influential and are highlighted in the corresponding tables.

Oxidative damage markers, SL concentrations, and SL-derived biomarkers were compared among experimental groups using one-way ANOVA after verification of variance homogeneity (Hartley test). The Kruskal–Wallis test was used for variables that did not follow a normal distribution. When significant differences were detected, group means were compared using Duncan’s multiple range test. Means not sharing a common letter were considered significantly different. In addition, a complementary analysis based on the 90th percentile (P90) of values obtained in control animals was performed on SL ratios using ROC curve analysis to evaluate the sensibility, specificity and accuracy of these biomarkers.

## Figures and Tables

**Table 1:** Hepatic sphingolipid concentrations in turkeys fed the Con diet or the FDZ diets ^1^.

Analyte	Con	FDZ	FDZ_Con2d	FDZ_Con4d
Sphingoid bases and derivates ^2^				
d18:0	1510 ± 351	1584 ± 292	1443 ± 284	1463 ± 471
d18:1	17,304 ± 3724	15,523 ± 2954	16,543 ± 2756	16,497 ± 4064
d20:0	248 ± 38.4	277 ± 45.8	230 ± 31.5	259 ± 52.1
m18:0	17.2 ± 4.8 a	**12.1 ± 2 b**	**9.2 ± 1.9 b**	**10.8 ± 2.2 b**
GluSo	547 ± 177	495 ± 209	579 ± 133	595 ± 152
LysoSM	263 ± 48.7	266 ± 49.3	312 ± 76.7	286 ± 56.5
18:0/2:0	40.3 ± 7.6 b	**55.2 ± 12.9 a**	44.9 ± 7.5 ab	**52.1 ± 9.5 a**
18:1/2:0	165 ± 31	**212 ± 58.6**	194 ± 46.7	**218 ± 46.3**
Dihydroceramides (DHCer) ^3^				
18:0/16:0	40.5 ± 9.5	39.7 ± 10.1	36.2 ± 6.1	40.7 ± 14.6
18:0/17:0	0.6 ± 0.2	0.7 ± 0.2	**0.8 ± 0.3**	0.7 ± 0.3
18:0/18:0	10.1 ± 3.5	9.8 ± 3.5	8 ± 2.6	9.7 ± 1.8
18:0/20:0	3.7 ± 1	4 ± 1.3	3.1 ± 1	3.2 ± 1
18:0/22:0	2.4 ± 1	2.4 ± 0.5	2.4 ± 0.4	2.3 ± 0.6
18:0/23:0	1.2 ± 0.4	1.4 ± 0.2	1.2 ± 0.3	1.1 ± 0.2
18:0/24:0	2.7 ± 0.7	3 ± 0.6	2.4 ± 0.6	2.7 ± 0.6
Ceramides (Cer) ^3^				
18:1/14:0	4.8 ± 1.3	**3.9 ± 0.8**	4.1 ± 0.9	4.2 ± 0.7
18:1/15:0	0.2 ± 0.1	**0.2 ± 0**	0.2 ± 0.1	**0.3 ± 0.1**
18:1/16:0	1116 ± 237 a	**838 ± 177 b**	**913 ± 156 ab**	971 ± 216 ab
18:1/17:0	15.6 ± 4.4 ab	**10.8 ± 3 b**	14.2 ± 4 ab	19 ± 7.9 a
18:1/18:0	183 ± 72.4	171 ± 49.2	149 ± 34.4	158 ± 47.9
18:1/18:1	1 ± 0.2 a	1 ± 0.2 a	**0.8 ± 0.3 b**	**0.6 ± 0.1 b**
18:1/20:0	109 ± 37.2	111 ± 32.3	108 ± 22.7	103 ± 26.7
18:1/22:0	196 ± 35.3	200 ± 36.4	179 ± 33.2	200 ± 40.3
18:1/22:1	18.9 ± 4.8 a	18.9 ± 4.2 a	16.5 ± 5 a	**11.5 ± 2.5 b**
18:1/22:2	13.5 ± 3.4	11.9 ± 2.2	12.5 ± 2.9	14.1 ± 4.4
18:1/23:0	60.1 ± 13.6	63.5 ± 12.3	65.6 ± 10.6	57.9 ± 10.6
18:1/23:1	10.4 ± 3	10.3 ± 2.5	9.6 ± 2	10.2 ± 4.5
18:1/24:0	137 ± 19.5	141 ± 20.3	140 ± 19.4	136 ± 23.6
18:1/24:1	229 ± 34.6 a	219 ± 38.7 ab	**194 ± 40.1 ab**	**180 ± 41.6 b**
18:1/24:2	92 ± 20.2	85.9 ± 14.4	86.6 ± 12.9	92.7 ± 21.9
18:1/25:1	19.6 ± 4.2	20.4 ± 3.6	19.6 ± 2.6	17 ± 6.4
18:1/26:0	1.1 ± 0.2	1.2 ± 0.1	1.1 ± 0.2	1.1 ± 0.2
18:1/26:1	5.2 ± 0.7 a	5.3 ± 0.6 a	4.9 ± 1 a	**4.4 ± 0.7 b**
18:1/26:2	3.6 ± 0.6	3.3 ± 0.5	**2.8 ± 0.8**	3.6 ± 1.1
Other ceramides ^3^				
m17:1/24:1	4.7 ± 1.3	5 ± 1	4.8 ± 0.7	5.3 ± 2.7
m18:0/22:0	0.1 ± 0.01	0.1 ± 0.01	0.1 ± 0.01	0.1 ± 0.01
m18:0/24:1	0.2 ± 0.02	0.2 ± 0.02	0.2 ± 0.05	0.2 ± 0.02
m18:1/16:0	7.4 ± 1.6	**5.9 ± 1.3**	**6.2 ± 1.1**	6.6 ± 1.5
m18:1/22:0	0.9 ± 0.2	0.9 ± 0.1	0.8 ± 0.2	0.9 ± 0.2
m18:1/24:1	3.4 ± 0.8	3.2 ± 0.6	2.7 ± 0.7	2.7 ± 1.2
18:2/16:0	2.2 ± 0.6 a	**1.8 ± 0.4 b**	1.6 ± 0.4 b	**1.4 ± 0.4 b**
18:2/22:0	1.1 ± 0.2 a	1.1 ± 0.2 a	1 ± 0.2 a	**0.7 ± 0.1 b**
18:2/24:1	0.7 ± 0.1 a	0.7 ± 0.2 a	0.6 ± 0.2 a	**0.4 ± 0.1 b**
t18:0/22:0	4.2 ± 0.9	3.9 ± 1.2	3.6 ± 1	5.3 ± 2.6
t18:0/24:0	3.6 ± 1.2	3.3 ± 0.6	4.1 ± 1.5	**3.6 ± 1.4**
t18:0/24:1	8.7 ± 2.3	7.1 ± 3	**6 ± 2.1**	7.3 ± 4.7
Monohexosylceramides (HexCer) ^3^				
Hex18:1/16:0	14.7 ± 3 a	**9.7 ± 1.4 b**	**9.4 ± 2.6 b**	13.4 ± 5.9 a
Hex18:1/18:0	1 ± 0.3	0.9 ± 0.2	**0.8 ± 0.2**	1 ± 0.3
Hex18:1/20:0	1.8 ± 0.7	1.5 ± 0.3	**1.3 ± 0.4**	1.7 ± 0.6
Hex18:1/22:0	28.6 ± 3	**31.1 ± 3**	29.1 ± 3	29.7 ± 4.5
Hex18:1/24:0	22.9 ± 5	23.5 ± 2.9	21.8 ± 5.3	25.5 ± 5.6
Hex18:1/24:1	8 ± 1.8	8.8 ± 1.3	7.3 ± 2.5	7.2 ± 1.1
Lactosylceramides (LacCer) ^3^				
Lac18:1/16:0	18.9 ± 7 a	14.6 ± 5.1 ab	**12.6 ± 4.5 b**	**12 ± 4.6 b**
Lac18:1/18:0	4.7 ± 1.2 a	4.7 ± 1.8 a	**3.5 ± 0.7 b**	**3.3 ± 1 b**
Lac18:1/20:0	30.7 ± 6.1	29.4 ± 7.7	**23.3 ± 6.5**	29.3 ± 7.7
Dihydrosphingomyelins (DHSM) ^3^				
SM18:0/16:0	18.6 ± 4.8	16.3 ± 3.4	**15 ± 3.9**	17.4 ± 3.8
SM18:0/18:0	10.5 ± 2.7	10.3 ± 1.2	8.9 ± 2.4	10 ± 2.8
SM18:0/20:0	4.6 ± 1.1	4.1 ± 0.9	3.4 ± 1.7	4 ± 1.2
SM18:0/22:0	19.1 ± 5	19.6 ± 3.3	**17.2 ± 4.5**	18.1 ± 5.5
SM18:0/23:0	**0.8 ± 0.2**	1 ± 0.1	**1 ± 0.2**	0.9 ± 0.2
SM18:0/24:0	3.4 ± 1.1	3.6 ± 0.8	3.1 ± 0.8	3.3 ± 1.1
SM18:0/24:1	1.2 ± 0.3	1.5 ± 0.4	1.2 ± 0.3	1.3 ± 0.3
Sphingomyelins (SM) ^3^				
SM18:1/14:0	0.1 ± 0 b	0.1 ± 0.1 b	0.1 ± 0 b	**0.2 ± 0.1 a**
SM18:1/15:0	0.1 ± 0.1 b	**0.1 ± 0 b**	0.1 ± 0 b	0.2 ± 0.1 a
SM18:1/16:0	127 ± 31.9 a	**86.5 ± 22.9 b**	**93.8 ± 19.3 b**	122 ± 30.8 a
SM18:1/17:0	5 ± 0.8 b	**4.3 ± 0.7 b**	4.7 ± 1.3 b	**6.3 ± 1.8 a**
SM18:1/18:0	134 ± 33.2	122 ± 20.8	117 ± 29.8	135 ± 41.1
SM18:1/20:0	51.1 ± 10.1	49.2 ± 7.2	46.6 ± 9.5	51.6 ± 15.4
SM18:1/22:0	590 ± 111	620 ± 119	589 ± 147	669 ± 259
SM18:1/22:1	2.9 ± 1.4	3.3 ± 1.4	2.7 ± 0.7	2.5 ± 1.1
SM18:1/22:2	0.7 ± 0.2	0.9 ± 0.3	1.1 ± 0.6	**1.1 ± 0.4**
SM18:1/23:0	52.3 ± 5.1	**60.3 ± 6.8**	53.7 ± 7.8	56.5 ± 14.3
SM18:1/23:1	1.5 ± 0.6	1.7 ± 0.6	1.7 ± 0.4	1.9 ± 0.7
SM18:1/24:0	68.1 ± 12.7	**79.2 ± 12.3**	75 ± 13	**85.5 ± 26.7**
SM18:1/24:1	46.6 ± 21.3	62.7 ± 23.9	55.7 ± 11.7	62.8 ± 23.7
SM18:1/24:2	9.9 ± 5.6	11.1 ± 5.1	11 ± 2.5	**16.2 ± 6.8**
SM18:1/24:3	0.5 ± 0.1	0.5 ± 0.1	0.5 ± 0.1	**0.6 ± 0.2**
SM18:1/25:0	3.4 ± 1.2	4 ± 1	4 ± 1	4.6 ± 1.6
SM18:1/25:1	2.4 ± 0.8 b	**3.2 ± 0.9 ab**	**3.1 ± 0.6 ab**	**3.8 ± 1.3 b**
SM18:1/25:2	0.8 ± 0.3	1 ± 0.2	0.9 ± 0.2	**1.2 ± 0.4**
SM18:1/26:0	0.7 ± 0.2	0.7 ± 0.1	0.7 ± 0.2	0.8 ± 0.3
SM18:1/26:1	0.5 ± 0.1	**0.6 ± 0.1**	**0.6 ± 0.1**	**0.6 ± 0.2**
SM18:1/26:2	0.7 ± 0.1	0.7 ± 0.1	0.7 ± 0.1	**0.9 ± 0.3**

^1^ Values were obtained from 10 animals per group and are expressed as mean ± SD. Groups were compared by one-way ANOVA or the Kruskal–Wallis test. When a significant difference was observed (*p* < 0.05), pairwise comparisons were performed. Different letters within the same row indicate statistically significant differences between groups (*p* < 0.05). Variables contributing to the PLS-DA model (Figure 1) are shown in bold. ^2^ Expressed as pmol/kg liver. ^3^ Expressed as nmol/kg liver.

**Table 2:** Distribution of turkeys and chickens exposed to the FDZ diet among the different classification groups.

	P90 ^1^	Threshold ^2^	Sensitivity (%) ^2^	Specificity (%) ^2^	Accuracy (%) ^2^
Turkey					
SaSo	0.094	0.093	57	90	65
DHCer C22–C24:C16	0.166	0.162	50	90	60
Cer C22–C24:C16	0.761	0.753	70	90	75
DHSM C22–C24:C16	1.743	1.433	67	80	70
SM C22–C24:C16	7.839	6.494	90	80	88
HexCer C22–C24:C16	4.662	4.583	90	90	90
Chicken					
SaSo	0.090	0.090	37	90	50
DHCer C22–C24:C16	0.187	0.134	70	60	68
Cer C22–C24:C16	1.528	1.523	40	90	53
DHSM C22–C24:C16	1.751	1.72	57	90	65
SM C22–C24:C16	9.987	9.749	73	90	78
HexCer C22–C24:C16	4.703	4.704	33	100	50

^1^ Percentile (P90) values of the ratios measured in the livers of control animals (*n* = 10). ^2^ ROC curve analysis of distribution of the animals in the exposed and unexposed groups at the P90 level. Sensitivity corresponded to the test’s ability to correctly identify true positive cases. Specificity corresponded to the test’s ability to correctly identify true negative cases. Accuracy was the proportion of individuals correctly classified by the test, including both positive and negative results.

**Table 3:** Plasma sphingolipid concentrations in turkeys fed the control diet or the FDZ diet ^1^.

Analyte	Con	FDZ	FDZ_Con2d	FDZ_Con4d
d18:0	18.5 ± 5.1 a	17.8 ± 7.8 a	**10.7 ± 2.7 b**	19.1 ± 9.8 a
d18:1	116 ± 15 a	**100 ± 25 a**	**78 ± 8 b**	117 ± 38 a
d18:0P	2009 ± 299 ab	**2123 ± 260 ab**	1693 ± 307 b	**2417 ± 696 a**
d18:1P	6369 ± 977 ab	**7157 ± 742 ab**	**6113 ± 1216 b**	**7733 ± 1982 a**
18:0/2:0	3.1 ± 0.9 b	**5.4 ± 1.1 a**	**4.4 ± 1.4 ab**	**4.2 ± 1.4 ab**
Sa1P:So1P	0.35 ± 0.02 a	**0.33 ± 0.02 ab**	**0.31 ± 0.03 b**	0.35 ± 0.03 a
18:1/16:0	1281 ± 267 a	**956 ± 184 b**	**894 ± 227 b**	**1068 ± 437 ab**

^1^ Values were obtained from 10 animals per group and are expressed as mean ± SD (pmol/L plasma). Groups were compared by one-way ANOVA or the Kruskal–Wallis test. When a significant difference was observed (*p* < 0.05), pairwise comparisons were performed. Different letters within the same row indicate statistically significant differences between groups (*p* < 0.05). Variables contributing to the PLS-DA model (Figure 4) are shown in bold. Results for all sphingolipids are presented in Table S2.

**Table 4:** Hepatic sphingolipid concentrations in chickens fed the control diet or the FDZ diet ^1^.

Analyte	Con	FDZ	FDZ_Con2d	FDZ_Con4d
d18:0 ^2^	921 ± 288 b	**1215 ± 389 ab**	1066 ± 240 ab	**1386 ± 340 a**
d18:1 ^2^	11,863 ± 4258 b	13,434 ± 3730 ab	13,381 ± 2842 ab	**16,819 ± 4795 a**
GluSo ^2^	245 ± 96 b	**313 ± 78 b**	**318 ± 107 b**	**482 ± 157 a**
LysoSM ^2^	152 ± 30 b	**178 ± 46 ab**	**176 ± 41 ab**	**222 ± 72 a**
18:0/16:0 ^3^	28.9 ± 5.5	**37.3 ± 10.6**	**33.3 ± 8.4**	**36.3 ± 8.4 a**
18:0/20:0 ^3^	0.29 ± 0.07 b	**0.46 ± 0.12 a**	**0.47 ± 0.15 a**	**0.42 ± 0.1 a**
18:0/22:0 ^3^	0.44 ± 0.16 b	**0.7 ± 0.24 a**	**0.58 ± 0.19 ab**	**0.63 ± 0.14 ab**
18:0/23:0 ^3^	1.21 ± 0.36 b	**1.78 ± 0.5 a**	1.22 ± 0.22 b	**1.48 ± 0.38 ab**
18:0/24:0 ^3^	2.57 ± 0.79 b	**3.48 ± 0.86 a**	2.6 ± 0.48 b	3.01 ± 0.63 ab
Sum of DHCer ^3^	41.1 ± 8.1 b	**55.1 ± 16.9 a**	**47.7 ± 11.1 ab**	**51.5 ± 10.7 ab**
Cer C22–24:C16	1.46 ± 0.09 ab	1.49 ± 0.08 ab	**1.54 ± 0.11 a**	1.39 ± 0.13 b
m17:1/24:1 ^3^	0.9 ± 0.31 b	0.95 ± 0.2 b	**2.31 ± 1.08 a**	**0.85 ± 0.19 b**
m18:1/22:0 ^3^	0.47 ± 0.06 b	0.50 ± 0.07 b	**0.61 ± 0.08 a**	0.47 ± 0.07 b
m18:1Cer C22-C24:C16	0.8 ± 0.12 ab	0.76 ± 0.11 ab	0.87 ± 0.11 a	0.7 ± 0.09 b
t18:0/24:1 ^3^	5.64 ± 2.41 b	5.82 ± 1.5 b	**8.28 ± 2.36 a**	6.53 ± 1.31 ab
Sum t18:0-Cer ^3^	10.5 ± 3.6 b	10.7 ± 2.6 b	**14.8 ± 3.9 a**	11.8 ± 3 ab
Hex18:1/18:0 ^3^	1.30 ± 0.47 b	**1.05 ± 0.32 b**	1.24 ± 0.43 b	**1.73 ± 0.61 a**
Lac18:1/20:0 ^3^	28.4 ± 4 a	**23.9 ± 2.3 b**	**24.2 ± 4.5 b**	29.6 ± 3.3 a
DHSM C22-C24:C16	1.5 ± 0.29 b	**1.76 ± 0.31 ab**	1.94 ± 0.38 a	1.63 ± 0.32 ab
SM C22-C24:C16	8.2 ± 1.9 b	**10.9 ± 2.5 a**	**11.7 ± 3.0 a**	**10.1 ± 1.9 ab**

^1^ Values were obtained from 10 animals per group and are expressed as mean ± SD. Groups were compared by one-way ANOVA or the Kruskal–Wallis test. When a significant difference was observed (*p* < 0.05), pairwise comparisons were performed. Different letters within the same row indicate statistically significant differences between groups (*p* < 0.05). Variables contributing to the PLS-DA model (Figure 5) are shown in bold. Results for all sphingolipids are presented in Table S3. ^2^ Expressed as pmol/kg liver. ^3^ Expressed as nmol/kg liver.

**Table 5:** Plasma sphingolipid concentrations in chickens fed the control diet or the FDZ diet ^1^.

Analyte ^2^	Con	FDZ	FDZ_Con2d	FDZ_Con4d
d18:1	59.9 ± 12 ab	**71.9 ± 22.2 b**	**46.7 ± 8 b**	54.1 ± 17.3 ab
d18:0P	663 ± 189 b	**852 ± 284 a**	**551 ± 119 b**	577 ± 131 b
d18:1P	2242 ± 603 b	**2809 ± 731 a**	**1687 ± 390 b**	1944 ± 532 b
18:0/22:0	48.5 ± 22.8 ab	62.4 ± 33.4 a	**33.6 ± 8.5 b**	**32.8 ± 11 b**
18:0/23:0	112.5 ± 48.1 ab	143.8 ± 59.9 a	**77.8 ± 26.1 b**	**80.7 ± 19.5 b**
18:0/24:0	228 ± 105 b	**336 ± 156 a**	181 ± 50 b	185 ± 57 b
Sum of DHCer	437 ± 202 b	**585 ± 231 a**	**336 ± 57 b**	373 ± 68 b
18:1/18:0	562 ± 203 a	**497 ± 152 ab**	**369 ± 103 b**	**393 ± 132 b**
18:1/18:1	58.1 ± 18.2 b	**91.8 ± 31 a**	60.9 ± 20.7 b	**74.4 ± 23.6 ab**
18:1/22:0	3182 ± 1039 a	3227 ± 811 a	**2395 ± 485 ab**	**2123 ± 797 b**
18:1/24:0	3906 ± 1320 ab	4332 ± 1266 a	**3173 ± 633 bc**	**2762 ± 708 c**
18:1/24:1	4573 ± 1257 a	4640 ± 1030 a	**3225 ± 618 b**	**2899 ± 1296 b**
18:1/24:2	1853 ± 558 a	**1516 ± 292 ab**	**1365 ± 135 b**	**1329 ± 407 b**
18:1/25:1	321 ± 68 ab	370 ± 99 a	**283 ± 29 b**	**264 ± 59 b**
18:1/26:0	229 ± 23 ab	**250 ± 37 a**	231 ± 21 ab	**210 ± 19 b**
18:1/26:1	210 ± 27 ab	**232 ± 38 a**	209 ± 16 ab	**184 ± 25 b**
Sum of Cer	18,360 ± 5085 a	18,502 ± 3958 a	**14,037 ± 2116 b**	**12,919 ± 3984 b**
Cer C22–24:C16	9.92 ± 1.45 b	**11.24 ± 1.68 a**	9.53 ± 0.79 b	**8.22 ± 1.11 c**
m18:1/22:0	10.71 ± 4.86 a	10.54 ± 1.85 a	**8.4 ± 2.71 ab**	**6.67 ± 3.08 b**
m18:1/24:1	65.1 ± 18.5 a	60.6 ± 14.4 a	**40.4 ± 7.3 b**	**39.4 ± 17.2 b**

^1^ Values were obtained from 10 animals per group and are expressed as mean ± SD. Groups were compared by one-way ANOVA or the Kruskal–Wallis test. When a significant difference was observed (*p* < 0.05), pairwise comparisons were performed. Different letters within the same row indicate statistically significant differences between groups (*p* < 0.05). Variables contributing to the PLS-DA model (Figure 7) are shown in bold. Results for all sphingolipids are presented in Table S4. ^2^ Expressed as pmol/kg plasma.

**Table 6:** Concentrations of *Fusarium* mycotoxins in the experimental diets ^1^.

	Turkey		Chicken	
Variable	Con	FDZ	Con	FDZ
Fumonisin B1	0.045	6.47	0.02	6.07
Fumonisin B2	0.01	1.42	<0.01	1.27
Fumonisin B3	<0.01	0.705	<0.01	0.62
Moniliformin	<0.1	0.23	<0.1	<0.1
Deoxynivalenol (DON)	0.21	2.3	0.225	2.72
DON-3-glucoside	0.025	0.31	0.025	0.265
15 Acetyl-DON	0.05	0.1	0.09	0.18
3 Acetyl-DON	<0.01	0.015	<0.01	0.025
Nivalenol	0.03	0.19	0.01	0.18

^1^ Results are expressed as mg/kg feed. Diacetoxyscirpenol, 15-monoacetoxyscirpenol, T-2 toxin, HT-2 toxin, T-2 tetraol, verrucarol, deepoxy-deoxynivalenol, fusarenon-X, roridin A, verrucarin A, alpha-zearalenol, beta-zearalenol, alpha-zearalanol, beta-zearalanol, ergocornine, ergocristine, ergocryptine, ergometrine, ergosine, ergotamine, aflatoxin B1, aflatoxin B2, aflatoxin G1, aflatoxin G2, ochratoxin A, ochratoxin B, alpha-ochratoxin, cyclopiazonic acid, citrinin, patulin, and sterigmatocystin were below the method limit of detection (LOD; 0.01 or 0.02 mg/kg, depending on the analyte). Tenuazonic acid was below the LOD of 0.05 mg/kg.

## Data Availability

The original contributions presented in this study are included in the article/Supplementary Materials. Further inquiries can be directed to the corresponding author.

## Supplementary Materials

The following supporting information can be downloaded at https://www.mdpi.com/article/10.3390/toxins18090371/s1, Table S1. Variables used to reveal oxidative damages in the liver of turkeys and chickens fed the Con diet or the FDZ diet; Table S2. Sphingolipids in the plasma of turkeys fed the Con diet or the FDZ diet; Table S3. Sphingolipids in the liver of chickens fed the Con diet or the FDZ diet; Table S4. Sphingolipids in the plasma of chickens fed the Con diet or the FDZ diet.

## Abbreviations

The following abbreviations are used in this manuscript:

CAT	Catalase
Cer	Ceramide
CerS	Ceramide synthase
Con	Control
DHCer	Dihydroceramide
DHSL	Dihydrosphingolipid
DHSM	Dihydrosphingomyelin
DON	Deoxynivalenol
FB	Fumonisin B
FDZ	FB, DON and ZEN
GSH	Glutathione reduced
GSSG	Glytathione oxidized
HexCer	Monohexosylceramide
LacCer	Lactosylceramide
LOAEL	Low observed adverse effect level
LOD	Limit of detection
MDA	Malondialdehyde
NOAEL	No observed adverse effect level
PLS-DA	Partial least squares discriminant analysis
Sa	Sphinganine
SL	Sphingolipid
SM	Sphingomyelins
So	Sphingosine
VIP	Variable importance in projection
ZEN	Zearalenone
